# Treatment Combining Focused Ultrasound with Gastrodin Alleviates Memory Deficit and Neuropathology in an Alzheimer's Disease-Like Experimental Mouse Model

**DOI:** 10.1155/2022/5241449

**Published:** 2022-01-13

**Authors:** Kaixuan Luo, Yuhong Wang, Wen-Shiang Chen, Xiangjun Feng, Yehui Liao, Shaochun Chen, Yao Liu, Chengde Liao, Moxian Chen, Lijuan Ao

**Affiliations:** ^1^School of Rehabilitation, Kunming Medical University, Kunming, Yunnan Province, China; ^2^Department of Physical Medicine and Rehabilitation, National Taiwan University Hospital & National Taiwan University College of Medicine, Taipei City, Taiwan; ^3^Department of Radiology, The Third Affiliated Hospital of Kunming Medical University, Yunnan Cancer Hospital & Cancer Center, Kunming, Yunnan, China

## Abstract

Alzheimer's disease (AD) is the most common type of dementia but lacks effective treatment at present. Gastrodin (GAS) is a phenolic glycoside extracted from the traditional Chinese herb—Gastrodia elata—and has been reported as a potential therapeutic agent for AD. However, its efficiency is reduced for AD patients due to its limited BBB permeability. Studies have demonstrated the feasibility of opening the blood-brain barrier (BBB) via focused ultrasound (FUS) to overcome the obstacles preventing medicines from blood flow into the brain tissue. We explored the therapeutic potential of FUS-mediated BBB opening combined with GAS in an AD-like mouse model induced by unilateral intracerebroventricular (ICV) injection of A*β*_1-42_. Mice were divided into 5 groups: control, untreated, GAS, FUS and FUS+GAS. Combined treatment (FUS+GAS) rather than single intervention (GAS or FUS) alleviated memory deficit and neuropathology of AD-like mice. The time that mice spent in the novel arm was prolonged in the Y-maze test after 15-day intervention, and the waste-cleaning effect was remarkably increased. Contents of A*β*, tau, and P-tau in the observed (also the targeted) hippocampus were reduced. BDNF, synaptophysin (SYN), and PSD-95 were upregulated in the combined group. Overall, our results demonstrate that FUS-mediated BBB opening combined with GAS injection exerts the potential to alleviate memory deficit and neuropathology in the AD-like experimental mouse model, which may be a novel strategy for AD treatment.

## 1. Introduction

Dementia, a syndrome characterized by dysfunction in memory, thinking, behavior, and the ability to perform daily activities, is affecting 50 million people globally. There are continuing nearly 10 million new cases each year. Alzheimer's disease (AD) is the most common type of dementia, contributing to 60-70% of cases [1], generating physical, psychological, social, and economic impacts on patients, families, and society. AD is a neurodegenerative and one of the protein-conformation diseases [2, 3], pathologically characterized by extracellular beta-amyloid (A*β*) deposition, intracellular aggregation of neurofibrillary tangles (NFTs), and extensive loss of neurons [4, 5]. Under the pathological conditions, A*β* promotes the increase of glycogen synthase kinase 3*β*, which phosphorylates tau into phosphorylated tau (P-tau) [6]. A*β* depositions also contribute to proinflammation responses such as activation of microglia and astrocyte, synaptic dysfunction, and abnormal cell death [7, 8].

There are symptom-relief medications for AD, but no curative approaches available at present [4, 9]. Several AD medications, including Cholinesterase inhibitors (ChEIs) and the N-methyl-d-aspartate (NMDA) antagonist memantine, have been approved by the US Food and Drug Administration (FDA). Despite the limited clinical benefit, side effects such as nausea, vomiting, diarrhea, and severe cardiovascular response have been reported [6, 9, 10]. Aduhelm (aducanumab) is directed at the underlying A*β* pathology of AD and has been approved by the FDA recently through the accelerated approval pathway. The clinical benefit, however, remains unclear [11]. The treatment of AD continues to be an intractable problem to modern medicine, and the development of new therapies meets current urgent needs.

Gastrodin (GAS) is a phenolic glycoside extracted from the traditional Chinese herb—Gastrodia elata—and is considered the primary active constituent of rhizoma gastrodiae. GAS was traditionally used as a therapeutic agent for ailments such as dizziness, headache, convulsions, hypertension, and cardiovascular diseases [12, 13]. Besides, studies have demonstrated the potential of treating AD with GAS both in vitro and in vivo. GAS may be beneficial for A*β* pathology and symptom of AD via antioxidative effect [14], anti-inflammatory effect [13], antiapoptosis effect [12], and reducing the activity of *β* [15] as well as *γ*-secretase [16]. GAS can pass through the BBB with limited permeability [17], which reduces its treatment efficiency despite the promising therapeutic potential for AD.

Barriers between circulation and tissue, such as the blood-brain and blood-cerebral spinal fluid barriers (BBB and BCSFB), are responsible for protecting the central nervous system (CNS) from pathogens as well as toxins. According to the statistics, 98% of small molecules whose size are less than 400 Da and almost 100% of large molecules whose size are above 500 Da cannot pass through the BBB, which hinders therapeutic agents into the brain and becomes an obstacle of CNS disease treatment [18]. Focused ultrasound (FUS), namely, the ultrasound that works in a focused way, is an early-stage, noninvasive method to open the blood-brain barrier locally, transiently, and safely, which facilitates delivery of anticancer drugs [19], gene [20], and immune cells [21] into the brain. Cavitation plays a vital role in BBB opening via FUS. Firstly, the microbubbles were usually preinjected into the blood vessel and circulated through the sonicated region. Then, the FUS activates microbubbles to grow, oscillate (stable cavitation), and even collapse (inertial cavitation), affecting the cellular structure and leading to the opening of tight junctions in BBB [22, 23]. Opening the BBB by FUS has exerted its therapeutic potential for AD [24, 25]. A study [26] provided evidence that BBB opening via MRI-guided FUS enhanced the delivery of intravenously administered antibodies, and *β*-amyloid (A*β*) plaque pathology was reduced in an AD mouse model. Subsequently, Jordão et al. [24] carried out transcranial FUS BBB opening in the TgCRND8 mouse model; a reduction of plaque pathology was observed without additional therapeutic agents administered. Endogenous antibodies are found to bind to A*β* plaques, and glia activation was enhanced, which may contribute to the internalization of A*β*. The therapeutic effect of combining BBB opening with GAS on brain disorders has been demonstrated before. A study shows an antiepileptic effect by elevating GAS concentration in the cerebral spinal fluid after a single focused shockwave treatment [27].

The present study was directed to investigate the therapeutic potential of the combined treatment (BBB opening via FUS and GAS administration) on AD. What is more, we try to explain the mechanism behind from a relatively new point of view, that is, from a perspective of the waste-cleaning function of the brain. We first explored the safety of BBB opening on male Kunming mice via FUS and microbubbles. The therapeutic effect of FUS-mediated BBB opening combined with GAS treatment was investigated in an A*β*_1-42_-induced AD-like experimental mouse model. Unilateral A*β*_1-42_ intracerebroventricular (ICV) injection was carried out on male Kunming mice to establish the AD-like model. BBB openings of the left hippocampus and intraperitoneal (i.p.) GAS injections were performed within treatment duration.

## 2. Materials and Methods

### 2.1. Animals

A total of 60 male Kunming mice (KM mice, weight, 28-30 g) were obtained from Kunming Laboratory Animal Center (Kunming, China) for use in the experiment. All mice were housed as five per cage in controlled temperature (24 ± 2°C) and humidity (50 ± 5%) on a 12 h reverse light/dark cycle with food and water ad libitum. All animal protocols were approved by the Animal Ethics Committee of Kunming Medical University (No. KMMU2019078).

### 2.2. Experimental Design

**Figure 1:**
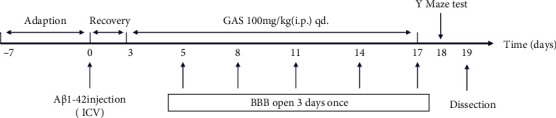
Timeline of the experimental protocol.

At the very beginning, we assessed the safety of BBB opening mediated by FUS. We observed its reversibility (*n* = 3 per time point) and performed H&E (*n* = 3 per time point) as well as TUNNEL (*n* = 3 per time point) staining of the targeted brain region before and 4 h, 24 h, 48 h, and 72 h after FUS sonication on normal KM mice. Influence on the safety by AD pathologic condition in the experimental procedure was hypothesized tiny and neglected. Then, mice were randomly allocated into five groups (*n* = 6 per group) after adaptation of 7 days: Group I, control: mice in this group were given ICV injection of saline rather than A*β*_1-42_ peptide and received placebo interventions within treatment duration; Group II, untreated: mice were intracerebroventricularly injected with A*β*_1-42_ peptide and given placebo treatment; Group III, GAS, all animals were intracerebroventricularly injected with A*β*_1-42_ peptide and received i.p. injection of GAS (100 mg/kg, qd.) daily; Group IV, FUS, mice were intracerebroventricularly injected with A*β*_1-42_ peptide and received FUS-mediated BBB opening every three days once for a total of 5 times; and Group V, FUS+GAS, mice that received ICV injection of A*β*_1-42_ peptide were given GAS treatment with a dose of 100 mg/kg once a day as well as FUS-mediated BBB opening every three days once for a total of 5 times. In general, mice were given ICV injection of A*β*_1-42_ peptide to establish an AD-like model at day 0 after one week of adaptation and were allowed a 3-day interval for recovery. Subsequently, treatments were given corresponding to grouping within the treatment duration (from days 3 to 17). Y-maze was carried out on day 18 to evaluate short-term memory, and mice were sacrificed with the left hippocampus collected for protein analysis on day 19 (Figure 1).

### 2.3. Safety of BBB Opening Mediated by FUS

We explored the reversibility of BBB opening mediated via FUS. To visualize the reversibility, 2% Evans Blue (EB, MilliporeSigma, Burlington, MA, USA) was injected via the tail vein (2.5 *μ*l/g) immediately after BBB opening mediated by FUS (the procedure is presented in 2.5) and allowed to circulate for 4 hours before sacrifice. The whole brain of mice was harvested. The upper surface and coronal plane of the sonicated brain region were pictured; then, the leakage of EB was observed.

H&E and TUNNEL staining was performed as a part of evidence regarding safety. For H&E staining, a set of HE dye solution (Servicebio G1003, Wuhan, Hubei, China) was used. Paraffin sections of the sonicated region were dewaxed through the following steps: incubate sections in 2 changes of xylene for 20 minutes each, 2 changes of 100% ethanol for 5 minutes each, and 75% ethanol for 5 minutes, then rinse with tap water. After dewaxing, sections were stained with hematoxylin solution for 3 to 5 minutes and rinsed with tap water. Then, sections were treated with hematoxylin differentiation solution and rinsed with tap water. Treat the sections with Hematoxylin Scott Tap Bluing, and rinse sections with tap water. Sections were dipped in 85% and 95% ethanol for 5 min, respectively. Then, stain sections with eosin dye for 5 min. Sections were dehydrated via 3 changes of 100% ethanol and 2 changes of xylene for 5 minutes each; finally, sections were sealed with neutral gum. We observed and photographed sections with a digital microscope. For TUNNEL staining, sections of the sonicated brain region were deparaffinized and rehydrated through a graded series of xylene to distilled water. To detect apoptosis of the sonicated brain tissue, a TUNNEL kit (Servicebio G1501, Wuhan, Hubei, China) was used to stain sections following the manufacturer's instructions. Sections were observed and photographed with a digital microscope.

### 2.4. Establishment of the AD-Like Experimental Mouse Model

We established an AD-like experimental mouse model via ICV injection of A*β*_1-42._ The surgical procedure was adapted from the literature [28]. A*β*_1-42_ (MilliporeSigma, Burlington, MA, USA) was dissolved in normal saline to prepare a stock solution with a final concentration of 1 *μ*g/*μ*l and then incubated at 37°C for 5 days to gain the fibrillized form. Mice were anesthetized by i.p. injection of 2% sodium pentobarbital (45 mg/kg). An incision of the scalp was made, and a total of 2 *μ*l incubated A*β*_1-42_ (1 *μ*g/*μ*l) was injected into the left lateral ventricle (coordinates from bregma: -0.94 mm anterior/posterior, 1.80 mm medial/lateral, and -2.40 mm dorsal/ventral) via a 5 *μ*l microsyringe with a speed of 0.2 *μ*l/min using a stereotaxic apparatus (RWD D02967, Shenzhen, Guangdong, China). The needle was left at the injected site for an additional five minutes, then withdrawn slowly (1 mm per minute) until complete removal from the brain. The incision of the scalp was sutured at last. Animal body temperature was maintained by an electric heating pad during and after the surgical procedure until the mouse got completely awake.

### 2.5. GAS Treatment and BBB Opening via FUS

**Figure 2:**
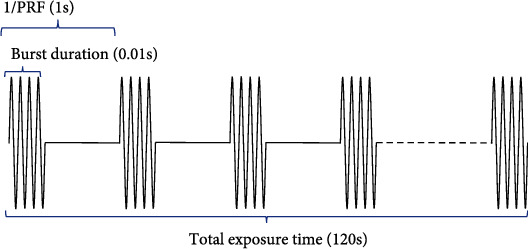
Schematic FUS parameters with 1 Hz pulse repetition frequency (PRF) and 1% duty cycle (DC).

**Figure 3:**
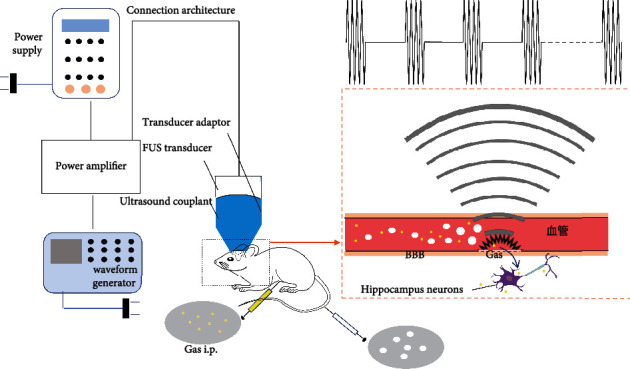
(a) Flow chart of combined treatment (BBB opening via FUS and GAS treatment). (b) Schematic diagram of BBB opening via FUS with the presence of microbubbles.

Mice were administrated GAS via i.p. injection once a day (100 mg/kg) for a total of 15 days (from day 3 to day 17). BBB opening mediated by FUS was performed three days once for a total of 5 times (day 5, 8, 11, 14, and 17) within treatment duration to prevent adverse events such as phlebitis of the caudal vein. To facilitate the opening of mice BBB, the fur on the scalp was removed before any treatment with a depilatory cream. A transducer adaptor was 3D printed to guide ultrasound focus to the left hippocampus. FUS transducer was loaded into the adaptor with ultrasound couplant filled in between and was preprepared before the FUS procedure. The transducer was driven by a waveform generator (RIGOL DG4202, Suzhou, Jiangsu, China) and a power amplifier (Mini-Circuits LZY-22+, New York, USA) to generate FUS. The total exposure time of sonication lasts for 120 seconds, and other parameters are as follows: fundamental frequency, 1 MHz; burst duration, 10 ms; pulse repetition frequency, 1 Hz (Figure 2); and microbubbles, SonoVue®, 2.5 *μ*l/g, with output voltage from the waveform generator set to 200 mV. FUS-mediated BBB opening or placebo operation was carried out immediately once GAS was injected. In summary, the mice were anesthetized with 1.5% isoflurane/oxygen using an isoflurane vaporizer (RWD R500, Shenzhen, Guangdong, China) and were fixed by using a mouse brain fixator with continuous anesthesia. Ultrasound couplant was filled between the transducer and mouse scalp, and the center of the transducer was aligned to the sonication site (coordinates from bregma: -2.70 mm anterior/posterior, 2.50 mm medial/lateral) via iron support. Microbubbles (SonoVue®) were diluted in normal saline then injected intravenously into the tail vein (2.5 *μ*l/g) 10 seconds prior to FUS sonication (Figure 3). Mice were put on an electric heating pad after the sonication to recover.

### 2.6. Y-Maze Test

Y-maze test was performed on day 18 to evaluate the working memory performance of mice. The apparatus is Y-shaped, consisting of 3 light-blue, opaque arms (30 cm long × 8 cm wide × 15 cm high), orientated at 120° from each other, connected by an intersection. A camera was fixed above the maze and videoed the activities of mice for analysis. One of the three arms was blocked to be the novel arm, and the arm that mice were placed in the beginning was recognized as the start arm. The training and testing section lasted 3 minutes with a 1-hour interval between each section. In the training section, the novel arm was blocked. The mice were introduced to the distal end of the start arm and allowed to explore the maze freely. In the testing section, the blockage of the novel arm was removed, and mice were introduced at the same position for testing. The time that mice spent in the novel arm was calculated.

### 2.7. Tissue Preparation

After the behavior test, mice were dealt with an overdose of 2% sodium pentobarbital i.p. and were perfused transcardially with prechilled saline (4°C). The left hippocampus was harvested (*n* = 5) rapidly for western blotting (WB) analysis and stored at −80°C immediately after collection until use.

### 2.8. Western Blotting Analysis

Tissue was weighed and dissected; then, radioimmunoprecipitation assay (RIPA) buffer (RIPA : phenylmethylsulfonyl fluoride (PMSF) = 1 ml : 10 *μ*l) was added. The tissue was homogenized via ultrasound and lysed on ice for 30 minutes, then centrifuged at 12000 r/min for 30 min at 4°C, and the supernatant was collected. The concentration of total protein was quantitated by an enhanced bicinchoninic acid (BCA) protein assay kit (Beyotime, China) and equalized to 30 *μ*g/10 *μ*l. Samples that contain a total of 30 *μ*g protein were resolved by 10% sodium dodecyl sulfate-polyacrylamide gel electrophoresis (SDS-PAGE) and transferred to polyvinylidene difluoride (PVDF) membranes (MilliporeSigma, Burlington, MA, USA). The membranes were blocked in 5% skim milk (taking Tris-buffered saline containing Tween 20 (TBST) as solvent) at room temperature, then incubated with appropriate primary antibodies at 4°C and were shaken gently. The primary antibodies used were monoclonal antibodies (mAbs) against A*β* (1 : 1000; Proteintech, China), tau (1 : 1000; Proteintech, China), BDNF (1 : 1000; Proteintech, China), synaptophysin (1 : 1000; Proteintech, China), *β*-actin (1: 2000; Santa Cruz Biotechnology, Dallas, TX, USA), polyclonal antibodies against P-tau (1 : 1000; Proteintech, China), AQP4 (1 : 1000; Proteintech, China), and PSD-95 (1 : 1000; Proteintech, China). Subsequently, we washed the membranes with TBST three times (15 minutes per time). Membranes were then incubated with horseradish peroxidase- (HRP-) conjugated goat anti-rabbit/mouse immunoglobulin G (IgG) secondary antibody (1 : 2000; Cell Signaling Technology, Danvers, MA, USA) for 2 hours at room temperature and washed three times with TBST. An enhanced chemiluminescence kit (ECL; Tanon, Shanghai, China) and Amersham Imager 600 (GE Healthcare Life Science, USA) were used to visualize and capture protein bands. A software—ImageJ (US National Institutes of Health, Bethesda, MD, USA)—was used to normalize the protein concentration, taking *β*-actin as reference.

### 2.9. Statistical Analysis

Results are presented as mean ± standard deviation (SD). SPSS version 20.0 (IBM Corp., Armonk, NY, USA) was used for all statistical analyses, and graphs were generated by GraphPad Prism software version 7.0 (GraphPad Software, Inc., San Diego, CA, USA). One-way analysis of variance (ANOVA) and two-factor ANOVA were used to process the time that mice spent in the novel arm and WB results. *P* < 0.05 was considered statistically significant.

## 3. Results

### 3.1. Safety of BBB Opening Mediated by FUS

**Figure 4:**
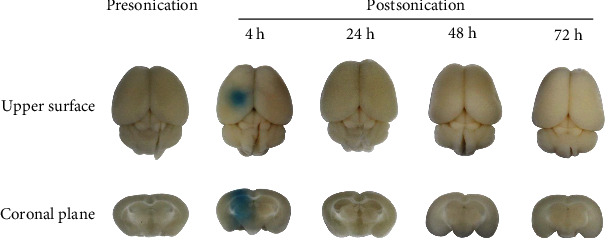
EB staining of mouse brain before/after FUS sonication. Obvious EB leakage is shown in the figure at 4 h after FUS sonication, while there is no EB staining presonication and at 24, 48, and 72 h postsonication.

We photographed the upper surface of mice's brains and the coronal plane of the sonicated region to observe the leakage of EB from the blood circulation. For mice that were sacrificed at 4 h after FUS sonication, obvious leakage in the brain parenchyma (including the targeted hippocampus) was observed, implying that BBB was effectively opened by FUS. However, there was no EB staining in mouse brains at 24 h, 48 h, and 72 h after FUS sonication (Figure 4), which indicates that FUS-mediated BBB opening in our experiment is reversible and the BBB closed within 24 h, avoiding the infectious risk of the central nervous system induced by long-term BBB opening.

**Figure 5:**
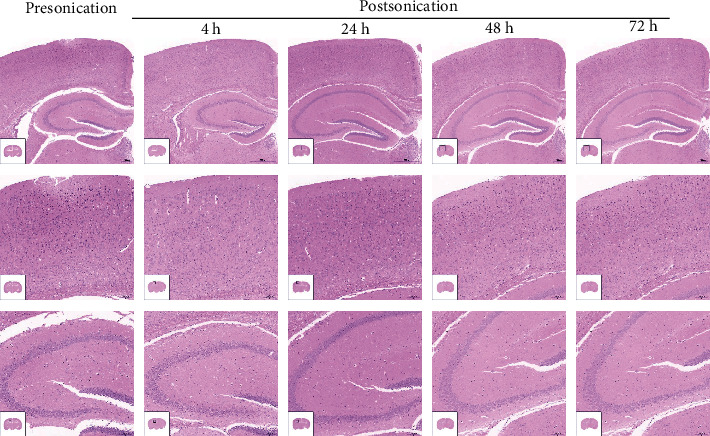
HE staining ((a) ×40, scale bar = 500 *μ*m; (b, c) ×100, scale bar = 100 *μ*m) shows that FUS-mediated BBB opening induced no edema, hemorrhage, or cell necrosis at 4, 24, 48, and 72 h postsonication.

**Figure 6:**
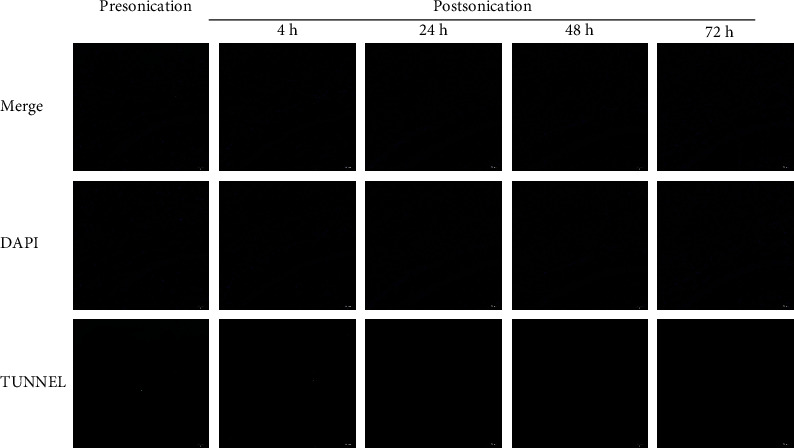
TUNNEL staining (×100, scale bar = 100 *μ*m) shows that there is no significant apoptosis at 4, 24, 48, and 72 h postsonication compared with presonication.

H&E staining showed no bleeding, cellular edema, nuclear fragmentation, or neutrophil infiltration in the sonicated region at 4 h, 24 h, 48 h, and 72 h after FUS-mediated BBB opening (Figure 5). TUNNEL staining of the targeted brain region at the same time points showed no significant apoptosis compared with brains that underwent placebo FUS treatment (Figure 6).

Collectively, the BBB of the sonicated brain region was effectively opened by FUS technology in our experiment and restored to the close state within 24 h, causing no bleeding and apoptosis until the next round of treatment.

### 3.2. FUS-Mediated BBB Opening Combined with GAS Treatment Increased the Time That AD-Like Mice Spent in the Novel Arm

**Figure 7:**
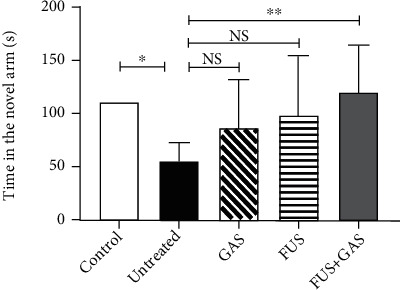
Protective effect of combined treatment (FUS+GAS) on short-term memory of AD-like mice. The time that AD-like mice spent in the novel arm of Y-maze was significantly shortened in comparison to control mice while prolonged via the combined treatment. Each symbol represents the mean ± SD; ^∗∗^*P* < 0.01 against untreated mice; ^∗^*P* < 0.05 against untreated mice. One-way ANOVA; *n* = 6 per group.

As shown in Figure 7, the time that untreated mice spent in the novel arm was significantly decreased in comparison to the control group (*P* < 0.05), implying that short-term memory was lesioned in ICV A*β*_1-42_-injected AD-like mouse model. On the other hand, FUS+GAS treatment statistically increased the time that mice spent in the novel arm compared with that of untreated mice (*P* < 0.01). For GAS and FUS groups, the mean time that mice spent in the novel arms was longer than that in untreated mice, but there was no significance (NS) between groups (*P* > 0.05).

### 3.3. FUS-Mediated BBB Opening Combined with GAS Treatment Reduced Contents of AD Biomarkers in the Observed (the Left) Hippocampus

**Figure 8:**
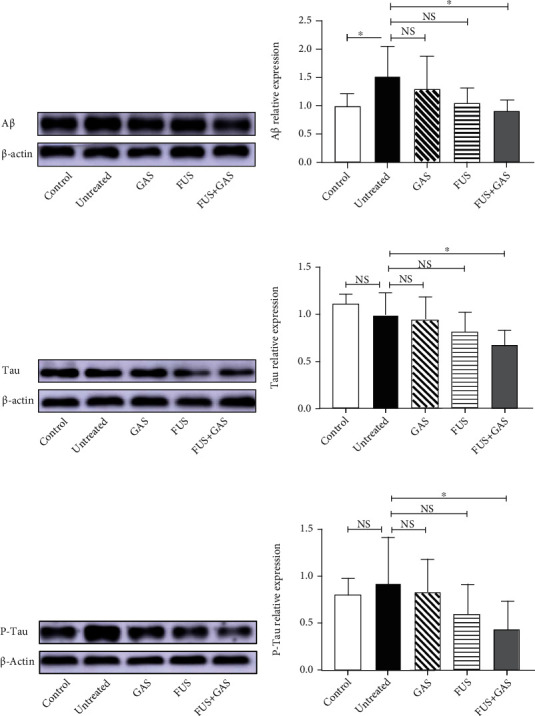
The effects of combined treatment (FUS+GAS) on the level of A*β* (a, b), tau (c, d), and P-tau (e, f) in the left hippocampus. The content of A*β* was increased markedly in the untreated group. Combined treatment reduced the content of A*β*, tau, and P-tau in the targeted hippocampus. Each symbol represents the mean ± SD; ^∗^*P* < 0.05 against untreated mice. One-way ANOVA; *n* = 5 per group.

We explored the level of A*β*, tau, and P-tau in the observed (the left) hippocampus of mice from different groups. For A*β*, the WB results (Figures 8(a) and 8(b)) revealed that ICV injection of A*β*_1-42_ significantly increased A*β* level in the observed hippocampus (*P* < 0.05), indicating the mouse model simulated the A*β* pathology of AD. In terms of the A*β* content and the behavioral performance, we successfully established an AD-like mouse model. FUS+GAS reduced the content level of A*β* in comparison to untreated mice (*P* < 0.05), while single GAS/FUS treatment failed to downregulate the A*β* level in the targeted hippocampus (*P* > 0.05). As for tau (Figures 8(c) and 8(d)) and P-tau (Figures 8(e) and 8(f)), combined treatment (FUS+GAS) also exerted an eliminative effect compared with the untreated group (*P* < 0.05). Similarly, single treatment (GAS/FUS treatment) was not enough to reduce the level of tau (*P* > 0.05) as well as P-tau (*P* > 0.05) in the targeted brain region. There was no statistical significance between control and untreated mice (*P* > 0.05) when it came to tau and P-tau.

### 3.4. FUS-Mediated BBB Opening Combined with GAS Treatment Upregulated the Expression of AQP4 in the Targeted Hippocampus

**Figure 9:**
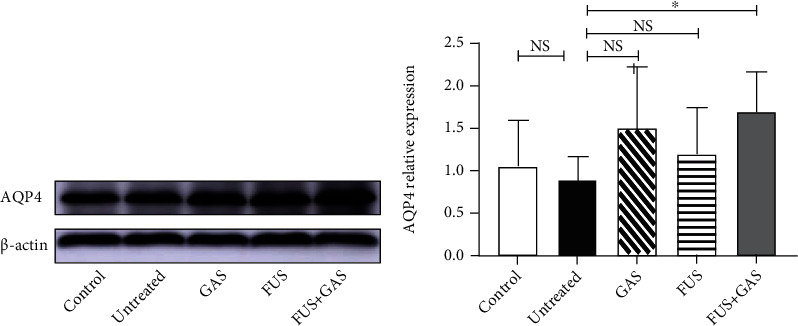
WB analysis of AQP4 level in sonicated hippocampus 15-days after interventions. The combined treatment of BBB opening via FUS and GAS remarkably increased the content of AQP4 in the targeted hippocampus, indicating a stronger waste-cleaning function. Each symbol represents the mean ± SD; ^∗^*P* < 0.05 against untreated mice. One-way ANOVA; *n* = 5 per group.

After a duration of 15-day intervention, FUS+GAS treatment upregulated the expression of AQP4 in the targeted hippocampus compared with untreated mice (*P* < 0.05, Figure 9). There was no significant difference between control and untreated, GAS and untreated, and FUS and untreated groups, respectively (*P* > 0.05).

### 3.5. FUS-Mediated BBB Opening Combined with GAS Treatment Upregulated BDNF, SYN, and PSD-95 Expressions in the Targeted Hippocampus

**Figure 10:**
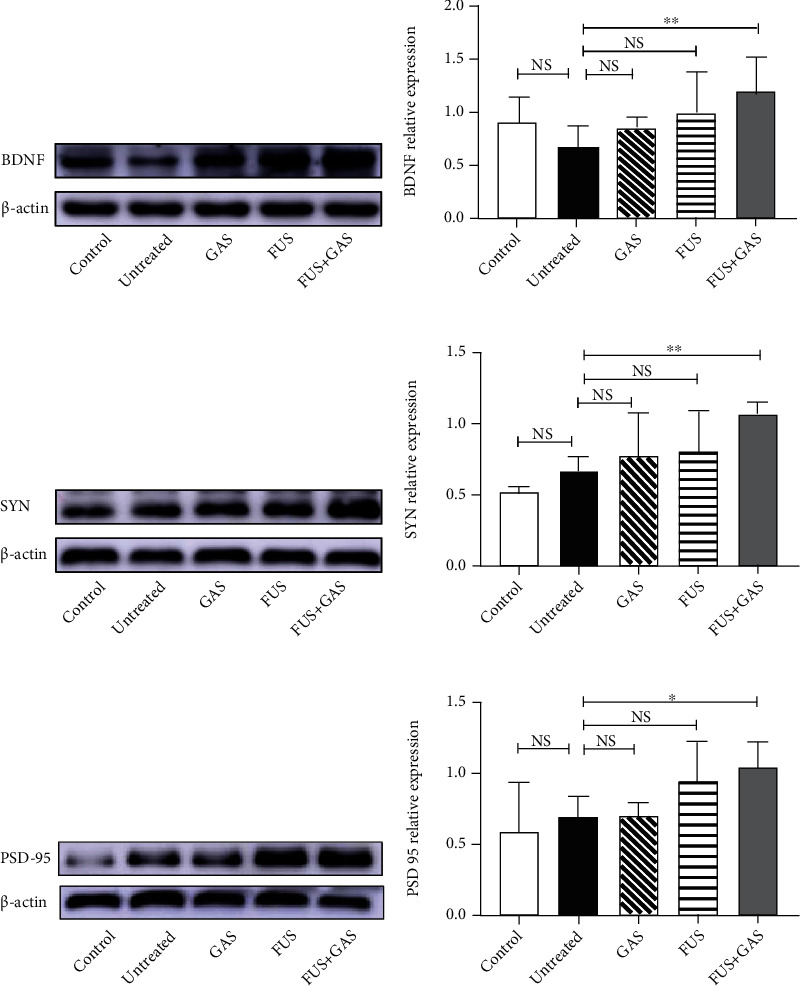
Relative concentrations of BDNF (a, b), SYN (c, d), and PSD-95 (e, f) in the sonicated hippocampus of all groups. Combined treatment (GAS+FUS) statistically upregulated the level of BDNF, SYN, and PSD-95 while single treatment (GAS/FUS alone) failed to. Each symbol represents the mean ± SD; ^∗∗^*P* < 0.01 against untreated mice; ^∗^*P* < 0.05 against untreated mice. One-way ANOVA; *n* = 5 per group.

In this study, the WB results showed a significant increase of BDNF level in the targeted hippocampus of the FUS+GAS group when compared with untreated mice (*P* < 0.01, Figures 10(a) and 10(b)). In contrast, statistical significance was absent between untreated mice and the remaining groups (*P* > 0.05). For SYN, the combined treatment (FUS+GAS treatment) exerted a promoting effect of expression in comparison to the untreated group (*P* < 0.01, Figures 10(c) and 10(d)). Single GAS/FUS treatment was not enough to induce such an effect of SYN level (*P* > 0.05). There was no statistical significance either between the control and untreated groups (*P* > 0.05). The results of PSD-95 were similar to those of SYN. Combined treatment rather than single GAS/FUS intervention upregulated the expression of PSD-95 in the targeted hippocampus (*P* < 0.05, Figures 10(e) and 10(f)). A significant change was absent between control and untreated mice (*P* > 0.05).

### 3.6. The Statistic Results Processed by Two-Factor ANOVA

We processed the time that mice spent in the novel arm and the WB results via two-factor ANOVA, trying to make it clear that whether the therapeutic effect of FUS+GAS is a result of additive effect. The *P* values are all above 0.05 as illustrated in Supplementary table 1.

## 4. Discussion

In this study, we demonstrated effective BBB disruption without evidence of tissue hemorrhage and apoptosis within the sonicated region on KM mice. EB staining confirmed the disrupted BBB area of the brain (including the left hippocampus) at 4 h after sonication. There was no EB leakage observed at 24 h, 48 h, and 72 h postsonication in the targeted brain region, indicating that the BBB restored automatically within 24 h. Furthermore, HE as well as TUNNEL staining at both early (4 h) and late time points (24, 48, and 72 h) revealed no blood corpuscle and apoptosis within the sonicated brain region. The BBB opening mediated by FUS in the present study is reversible and well tolerated without evidence of tissue damage.

Based on these experimental results, we further reported that FUS-mediated BBB opening combined with GAS treatment rather than single GAS/FUS intervention has multiple anti-AD effects on an AD-like experimental mouse model. We established the model successfully via ICV injection of A*β*_1-42_, in which the time that mice stayed in the novel arm of Y-maze is statistically shortened, and the A*β* level increased in the observed hippocampus. The combined treatment of BBB opening via FUS and GAS alleviates neuropathology of the AD-like mice by reducing the content of A*β* in the targeted hippocampus and attenuating the level of tau as well as P-tau in the same region.

A*β*, tau, and P-tau are biomarkers of AD and have been explored as diagnostic markers in blood and cerebrospinal fluid [29]. In AD, A*β* fibrils polymerize into insoluble amyloid fibrils that aggregate into senile plaques, which activate kinases, leading to hyperphosphorylation of tau and its polymerization into insoluble neurofibrillary tangles. The plaques and neurofibrillary tangles activate microglia and promote local inflammation, contributing to neurotoxicity [4]. Several studies take inhibiting A*β* synthesis as a therapeutic strategy for AD. It is well learned that GAS has the potential to suppress the activities of *β*-secretase (BACE) [15, 16] and *γ*-secretase [16]. Both secretases facilitate the synthesis of A*β*. Lim et al. reported decreased A*β* aggregation as well as the inhibition of BACE activity in A*β*-injected AD-like mice under the treatment of bojungikgi-tang (a traditional herbal formula), which ameliorates memory impairment and protects neurons [30]. In the present study, we found that not only A*β* but also tau as well as P-tau were markedly decreased under the combined treatment. The possible mechanism, an enhanced waste-cleaning function of the brain, is much less reported. Evidence suggests that abnormal aggregation of tau, combined with its decreased clearance, intensifies neurotoxicity in AD [31]. The imbalance of production and clearing of substances such as A*β* and tau is closely linked to the progression of AD pathology [31, 32]. The paravascular pathways in which AQP4 plays an important role are largely involved in the waste-cleaning effect [33]. Studies have shown that the exogenous A*β* which was injected into the brain can be cleared via the paravascular pathways [34, 35]. Accumulated evidence has shown that AQP4 is involved in the pathogenesis of AD. AQP4 is the most extensively expressed aquaporin on astrocytic endfeet in the brain [36], functions as a water channel, and is of great significance in maintaining brain homeostasis [37]. AQP4 is vital for waste clearance including A*β*. In the AQP4-null mice, 55% of A*β* clearance was blocked [35]. Another research demonstrated that tau can be cleaned from the brain through the paravascular pathway too, and the deletion of the AQP4 gene led to decreased waste-cleaning function, and elevated P-tau level in traumatic brain injury mice, increased axonal degeneration, neuroinflammation, and exacerbation of posttraumatic cognitive deficits were observed [33]. In our study, the pronounced elevation of AQP4 level was detected in the targeted hippocampus after 15-day combined treatment in AD-like mouse, implying that FUS-mediated BBB opening combined with GAS treatment enhanced the waste-cleaning function of the brain, which may explain the neuropathological improvement of AD-like mice. In this study, we find remarkable therapeutic efficiency of combined treatment rather than single GAS/FUS intervention on AD-like mice, implying that a combination of BBB opening via FUS and GAS treatment may be an advisable strategy to deal with AD.

AD is characterized by aggravating cognitive deficits [38], which benefit little from current medications. One of the vital findings in the present study is that FUS+GAS treatment prolonged the time that AD-like mouse spent in the novel arm as measured by the Y-maze test, indicating that FUS-mediated BBB opening of the targeted (the left) hippocampus combined with GAS treatment exerts an ameliorative effect on short-term memory function in the AD-like mouse model. It has been suggested that the right hippocampus plays a more important role in spatial-related short-term memory in respect of human [39], and there have been lasting explorations about functional lateralization of the hippocampus in rodents. Results from Sakaguchi and Sakurai suggest that the bilateral hippocampus of Wistar albino rats is involved in short-term memory. The right hippocampus plays a facilitating role while the left exerts the opposite, that is, a suppressive effect [40]. It appears [41] that when it comes to short-term memory, the right hippocampus plays a predominant role. The results from Shipton *et al.* [42], however, provide evidence that an intact left hippocampus is essential for the short-term memory. The study reported that unilateral silencing of either the left or right CA3 was sufficient to impair short-term memory (as measured by Y-maze and T-maze), whereas the left rather than the right CA3 silencing impaired performance on an associative spatial long-term memory task, suggesting a significant role of the left hippocampus on both long- and short-term memory, which may explain the discovery in our study; that is, FUS-mediated BBB opening of the left hippocampus combined with GAS treatment improved short-term memory function as measured by Y-maze.

Studies have revealed that the loss of synapses is the most relevant neurobiological basis of AD's cognitive impairment [43]. In AD, disabled plasticity impacts negatively on synaptic remodeling, axonal sprouting, neurogenesis, synaptogenesis, and long-term potentiation (LTP) [44], which enhances the efficiency of synapses and is thought to underlie memory and learning [45]. The hippocampus is a brain area critical for learning and memory, which is well-established vulnerable to damages such as synapse loss at the early age of AD [46, 47]. To explore whether the combined treatment has an impact on plasticity of the sonicated hippocampus, we evaluated the level of BDNF, SYN, and PSD-95 via WB.

As an important neurotrophin, BDNF is highly expressed in the brain and exerts vital effects on regulating synapses both structurally and functionally. BDNF is an ideal and essential regulator of cellular processes underlying cognition [45]. Numerous studies have demonstrated the critical role of BDNF in terms of hippocampal LTP. Deletion of the BDNF gene in mice causes impaired LTP, which was rescued by recombinant BDNF [48, 49]. SYN is the most abundant integral membrane protein of small synaptic vesicles, constituting 6% to 8% of the synaptic vesicle membrane protein [50]. Involved in the secretion, recycling of synaptic vesicles, and neurotransmitter releasing, SYN continues to be the most widely used marker for synapse density [51], and a great number of papers quote SYN as a synaptic marker [44, 51]. As a postsynaptic marker [44], PSD-95 is an abundant scaffold protein of postsynaptic density, regulates synaptic transmission, and plays an important role in synaptic plasticity, learning, and memory [52].

Our results showed that the combined treatment of FUS-mediated BBB opening and GAS administration remarkably elevated the level of a neurotrophin (BDNF), a presynaptic marker (SYN), and a postsynaptic marker (PSD-95) in the targeted hippocampus, indicating a BDNF-stimulating effect and better neuroplasticity resulting from the combined intervention, which may be the underlying mechanism of behavioral improvement that AD-like mice presented in the Y-maze. In our study, the level of SYN and PSD-95 from control to untreated mice seems to be a rising trend, which may be possibly explained by the transient adaptive synaptic response in the pathologic process of AD [53, 54].

Gastrodia elata Blume (Orchidaceae) has been long used for its anticonvulsant, analgesic, and sedative effect in countries such as China [55]. As a phenolic glycoside extracted from traditional Chinese herb, Gastrodia elata, GAS is a main active constituent of rhizoma gastrodiae. The action mechanism of GAS has been studied for more than 40 years since its isolation in 1978 [17]. Investigations reveal that GAS suppresses *γ*-aminobutyric acid (GABA) transaminase to increase GABA concentration [56] and exerts antioxidant and antiapoptotic effect [17], benefiting epilepsy. What is more, by inhibiting the increase of extracellular glutamate level and blocking the elevation of intracellular Ca^2+^ and neuronal NO synthesis, GAS generates a neuroprotective action [57]. The antiamnestic effect has been explored as well [58, 59]. By normalizing the serotoninergic system [60] and the dopaminergic system [61], GAS ameliorates memory deficits in 3,3′-iminodipropionitrile-induced rats. Our present study demonstrates the memory protective effect of BBB opening via FUS combined with GAS treatment in A*β*_1-42_-induced AD-like mouse, possibly via the BDNF-stimulating and neuroplasticity-promoting effect.

In summary, our results reveal that FUS-mediated BBB opening combined with GAS treatment reduces the content of A*β*, tau, and P-tau in the targeted hippocampus in an ICV A*β*_1-42_-injected AD-like experimental mouse model possibly because of the elevated content of AQP4, which implies a stronger waste-cleaning function. Results demonstrate that the combined treatment upregulated the level of BDNF, SYN, and PSD-95, which contribute to short-term memory improvement in the AD-like experimental mouse model. Exerting the potential to alleviate memory deficit and neuropathology of the AD-like mouse model, FUS-mediated BBB opening combined with GAS treatment may be a novel strategy for AD treatment.

## 5. Limitations

There are several limitations in our study. Firstly, we established an AD-like mouse model via ICV injection of A*β*_1-42_ and explored the anti-AD effects of BBB opening via FUS combined with GAS treatment. The therapeutic effect needs to be verified further on a transgenic model such as APP/PS1 mice. Moreover, we observed that the levels of AQP4, BDNF, SYN, and PSD-95 were elevated after the combined intervention, while the mechanisms by which it affects the expressions of the above proteins are unclear. Investigations are needed, and several issues are required to be addressed further. Given the view of functional lateralization of the hippocampus, BBB opening of the right hippocampus may be carried out to see whether there is any difference in terms of short-term or long-term memory outcome. Although the results suggest that combined treatment of BBB opening via FUS and GAS treatment exerts therapeutic potential of AD treatment in this study, the present statistic failed to clarify whether the therapeutic effect is a result of additive effect induced by BBB opening mediated by FUS combined with GAS treatment (as illustrated in supplementary table 1) possibly owing to influence factors such as sample size, dose, and frequency of GAS administration, which should be confirmed in the next-step study with a refined design.

## 6. Conclusions

The combined treatment of BBB opening by FUS and GAS exerts a memory protective effect in the A*β*_1-42_-induced AD-like mouse model. Moreover, the combined treatment alleviates neuropathology; the content of A*β*, tau, and P-tau is reduced possibly via a powerful waste-cleaning effect induced by the upregulation of AQP4. The combination of BBB opening via FUS and GAS treatment may be an advisable strategy to deal with AD.

## Data Availability

The data supporting the findings of present study is available from the corresponding author upon request.

## Abbreviations

AD: Alzheimer's disease
AQP4: Aquaporin-4
A*β*: Beta-amyloid
BACE: *β*-Secretase
BBB: Blood-brain barrier
BDNF: Brain-derived neurotrophic factor
ChEIs: Cholinesterase inhibitors
FUS: Focused ultrasound
GABA: *γ*-Aminobutyric acid
GAS: Gastrodin
ICV: Intracerebroventricular
KM: Kunming
NFTs: Neurofibrillary tangles
NMDA: N-Methyl-d-aspartate
PSD-95: Postsynaptic density protein 95
P-tau: Phosphorylated tau
SYN: Synaptophysin.

## Supplementary Materials

Supplementary Materials Supplementary table 1: the statistic results processed by two-factor ANOVA.
