# Supplementary material for: Treatment Combining Focused Ultrasound with Gastrodin Alleviates Memory Deficit and Neuropathology in an Alzheimer's Disease-Like Experimental Mouse Model
Source: Neural Plast. 2022 Jan 13;2022:5241449. doi: 10.1155/2022/5241449 (PMC8776436; doi:10.1155/2022/5241449)
Supplement: Supplementary Materials — Supplementary table 1: the statistic results processed by two-factor ANOVA. [file 5241449.f1.doc]

**Supplementary table 1 The statistic results processed by two-factor ANOVA**

| Items | Time in novel arms | Aβ relative expression | tau relative expression | P-tau relative expression | AQP4 relative expression | BDNF relative expression | SYN relative expression | PSD-95 relative expression |
| --- | --- | --- | --- | --- | --- | --- | --- | --- |
| N of intervention (GAS) | 12 | 10 | 10 | 10 | 10 | 10 | 10 | 10 |
| N of intervention (FUS) | 12 | 10 | 10 | 10 | 10 | 10 | 10 | 10 |
| *F* value for GAS*FUS | 0.076 | 0.047 | 0.314 | 0.051 | 0.058 | 0.000 | 0.624 | 0.271 |
| *P* value for GAS*FUS | 0.785 | 0.832 | 0.583 | 0.825 | 0.813 | 0.989 | 0.441 | 0.610 |
